# Revolution in acute ischaemic stroke care: a practical guide to mechanical thrombectomy

**DOI:** 10.1136/practneurol-2017-001685

**Published:** 2017-06-24

**Authors:** Matthew R B Evans, Phil White, Peter Cowley, David J Werring

**Affiliations:** 1 Stroke Research Centre, Department of Brain repair and Rehabilitation, University College London Institute of Neurology, London, UK; 2 Stroke Research Centre, Institute of Neuroscience and Newcastle University Institute for Ageing, Newcastle Upon Tyne, UK; 3 Neuroradiological Academic Unit, University College London Institute of Neurology, London, UK

**Keywords:** stroke, thrombolysis, mechanical, thrombectomy

## Abstract

Rapid, safe and effective arterial recanalisation to restore blood flow and improve functional outcome remains the primary goal of hyperacute ischaemic stroke management. The benefit of intravenous thrombolysis with recombinant tissue-type plasminogen activator for patients with severe stroke due to large artery occlusion is limited; early recanalisation is generally less than 30% for carotid, proximal middle cerebral artery or basilar artery occlusion. Since November 2014, nine positive randomised controlled trials of mechanical thrombectomy for large vessel occlusion in the anterior circulation have led to a revolution in the care of patients with acute ischaemic stroke. Its efficacy is unmatched by any previous therapy in stroke medicine, with a number needed to treat of less than 3 for improved functional outcome. With effectiveness shown beyond any reasonable doubt, the key challenge now is how to implement accessible, safe and effective mechanical thrombectomy services. This review aims to provide neurologists and other stroke physicians with a summary of the evidence base, a discussion of practical aspects of delivering the treatment and future challenges. We aim to give guidance on some of the areas not clearly described in the clinical trials (based on evidence where available, but if not, on our own experience and practice) and highlight areas of uncertainty requiring further research.

## Introduction

In the UK, stroke is the most common serious neurological disease (incidence 115–150 per 100 000 population)^1 2^ and a leading cause of death;^3^ there are more than 1.2 million stroke survivors,^4–7^ of whom more than 50% have a disability.^8^ Improving outcome from stroke is thus a key healthcare priority. About 80% of acute strokes are ischaemic,^9^ mainly from large vessel occlusion due to either artery-to-artery embolism or cardiac embolism. Early treatment is critical to rescue potentially salvageable tissue (‘time is brain’)^10 11^: safe, rapid and effective arterial recanalisation to restore blood flow and improve functional outcome remains the primary goal of hyperacute ischaemic stroke management.^12^

Until recently, the only licensed treatment for acute ischaemic stroke was intravenous thrombolysis with recombinant tissue-type plasminogen activator (IV r-tPA). However, since November 2014, nine positive randomised controlled trials of mechanical thrombectomy have been published (table 1), leading to a revolution in the care of patients with acute ischaemic stroke due to large vessel occlusion in the anterior circulation. The efficacy of this treatment is unmatched by any previous therapy in stroke medicine, with a number needed to treat of less than 3 for improved functional outcome.^13^ With effectiveness shown beyond any reasonable doubt, the key challenge is how to implement safe and effective services accessible to the patients who need it.

**Table 1:** Details of the nine positive thrombectomy trials

Trial	Trial dates	Centres	Participants	Primary outcome measure	Age (years)	Onset of symptoms		NIHSS
						IV r-tPA	MT	
MR CLEAN^35^	2010–14	16	502	mRS at 90 days	≥18	≤4.5	≤6	>1
REVASCAT^36^*	2012–14	4	206	mRS at 90 days	18–80‡	≤4.5	≤8	>5
EXTEND 1A^37^†	2012–14	10	70	Reperfusion at 24 hours, NIHSS at day 3	≥18	≤4.5	≤6	No restriction
SWIFT-prime^38^†	2012–14	39	196	mRS at 90 days	18–80	≤3.5	≤6	8–29
ESCAPE^39^†	2013–14	22	316	mRS at 90 days	≥18	≤4.5	≤12	>5
THRACE^40^†	2010–15	26	402	mRS≤2 at 90 days	18–80	≤4	≤5	10–25
THERAPY^41^*	2012–14	36	108	mRS≤2 at 90 days	18–85	≤4.5§	≤8¶	>7
PISTE^42^	2013–15	10	65	mRS≤2 at 90 days	≥18	≤4.5	6	No restriction
EASI^43^*	2013–14	1	77	mRS≤2 at 3 months	≥18	<3	≤6	>7**

*Enrolment was halted early after positive results for thrombectomy were reported from other similar trials.

†Trial stopped early due to efficacy.

‡After enrolling 160 patients, inclusion criteria were modified to include patients up to the age of 85 years with an ASPECTS >8.

§Three-hour limit if patient>80 with diabetes, previous stroke, previous anticoagulation and NIHSS>25.

¶Revised protocol reduced cut-off to 5 hours.

**Or the presence of clinical imaging mismatch, and suspected or proven occlusion of the M1 or M2 segments of the middle cerebral artery, supraclinoid internal carotid artery or basilar artery.

IV r-tPA, intravenous thrombolysis with recombinant tissue-type plasminogen activator; mRS, modified Rankin Scale; MT, mechanical thrombectomy; NIHSS, National Institutes of Health Stroke Scale.

## Background: the evidence

### Intravenous recombinant tissue-type plasminogen activator (alteplase) and its limitations

Intravenous recombinant tissue-type plasminogen activator (IV r-tPA) 0.9 mg/kg is licensed for use in the UK up to 4.5 hours post symptom onset.^14 15^ In a meta-analysis of 6756 patients in nine randomised trials comparing alteplase with placebo or open control, treatment within 3 hours resulted in good outcome for 259 (32.9%) of 787 patients who received alteplase compared with 176 (23.1%) of 762 who received control (OR 1.75, 95% CI 1.35 to 2.27).^13^ Rapid delivery of intravenous thrombolysis after stroke onset is crucial: the number needed to treat for an excellent outcome roughly doubles from 5 (for treatment within 90 min) to 9 (when treatment is given at 3.0–4.5 hours).^13^ However, the *relative* benefit of IV r-tPA appears to be consistent regardless of age or stroke severity.^13^ Stroke services burgeoned around intravenous thrombolysis, with development of hyperacute stroke units, pathways and protocols, emergency stroke teams and public awareness campaigns, in order to allow populations to access this effective treatment as quickly as possible.

However, the benefit of IV r-tPA for patients with severe stroke due to large artery occlusion is variable, due largely to failure of early recanalisation (generally less than 30% for carotid, proximal middle cerebral artery or basilar artery occlusion).^16^ More importantly, there is a good clinical outcome in only ~25% of patients (at best) with proximal anterior circulation or basilar artery occlusion.^17^ Important independent risk factors predicting poor outcome post intravenous thrombolysis are the length^18–22^ and location^23 24^ of the arterial thrombus. This lack of efficacy of the only licensed treatment led to efforts to remove larger arterial clots using intra-arterial techniques, initially using lytic but then mechanical means.

### Intra-arterial versus intravenous thrombolysis

The PROACT II trial randomised 180 patients with acute ischaemic stroke due to proven occlusion of the middle cerebral artery and without haemorrhage or major early infarction signs on CT scan to heparin and intra-arterial pro-urokinase or heparin alone; 40% in the intervention arm achieved a good outcome compared with 25% in the control arm.^25^ This promising endovascular approach led to the development of mechanical thrombectomy.

### Mechanical thrombectomy

The introduction of mechanical intra-arterial clot retrieval into clinical practice heralds a new age in the acute management of ischaemic stroke for patients with acute large artery occlusive stroke. The Food and Drug Administration gave clearance to the first endovascular device: Merci Retrieval System (MERCI), in August 2004.^26^ The MERCI trial^27^ demonstrated a recanalisation rate (including the basilar artery) of 46% by MERCI device alone and 60.8% when combined with adjuvant intra-arterial recombinant tissue-type plasminogen activator. Intracranial haemorrhage occurred in 7.8%. The MultiMERCI trial^28^ used a later-generation MERCI device and demonstrated 69.5% recanalisation after device and adjunctive lytic (intra-arterial or intravenous) with favourable clinical outcomes in 34%, but there was no control medical therapy group.

Optimism about thrombectomy was diminished when three early randomised controlled trials published in 2013^29–31^ failed to show improved efficacy of endovascular clot retrieval compared with intravenous thrombolysis. However, the study designs were criticised because of the following: limitations in patient selection (in one of the studies,^29^ documented large vessel occlusion was not required), use of older technology (mainly first-generation clot retrieval devices) and a long delay from stroke onset to intervention. Nevertheless, in a *post hoc* subgroup analysis of those with CT angiogram-proven large vessel occlusion, there was a statistical benefit from endovascular treatment within 90 min of IV r-tPA.^32^

New-generation stent retriever devices (the Solitaire FR Revascularisation Device and Trevo ProVue Retriever) were studied in two small randomised controlled trials,^33 34^ which showed significantly better recanalisation compared with the older MERCI device; indeed, the SWIFT study^33^ was stopped early due to significantly better recanalisation with Solitaire (83% vs 48.1% with MERCI), as well as reduced mortality at 3 months (17.2% vs 38.2%) and better neurological outcome at 90 days.

Everything changed with the publication, in rapid succession, of nine landmark randomised controlled trials,^35–43^ testing new-generation stent retriever devices (between December 2010 and February 2015), which showed the consistently clear superiority of endovascular clot retrieval over standard medical care alone in reducing disability at 90 days in patients with ischaemic stroke due to anterior circulation large vessel occlusion, as measured by the modified Rankin Scale (mRS; the primary outcome measure). The first study to report was the Multicenter Randomised Clinical Trial of Endovascular Treatment for Acute Ischaemic Stroke in the Netherlands (MR CLEAN),^35^ with subsequent studies all stopped early due to efficacy, loss of equipoise or both (and it should be noted that stopping early might have caused the later trials to overestimate the effect size of the treatment). Tables 1–3 summarise some key features of these studies. Note that, unlike the previous neutral trials, these all selected patients with proven large artery occlusion using CT angiography and mostly randomised patients within 6 hours of stroke onset (table 1).

**Table 2:** Treatment details for participants in each cohort

Trial	Mechanical thrombectomy cohort				IV r-tPA cohort			
	Treatment	n	Age (±SD)	Median NIHSS (IQR)	Treatment	n	Age (±SD)	Median NIHSS (IQR)
MR CLEAN^35^	±IV r-tPA + MT ± (IA r-tPA or intra-arterial urokinase)	233	65.8 (54.5–76)‡	17 (14–21)	±IV r-tPA	267	65.7 (55.5–76.4)‡	18 (14–22)
REVASCAT^36^*	±IV r-tPA + M.T.	103	65.7 (±11.3)¶	17 (14–20)	±IV r-tPA	103	67.2 (±9.5)¶	17 (12–19)
EXTEND 1A^37†^	IV r-tPA ± M.T.	35	68.6 (±12.3)¶	17 (13–20)	IV r-tPA	35	70.2 (±11.8)¶	13 (9–19)
SWIFT-prime^38^†	IV r-tPA ± M.T.	98	65.0 (±12.5)¶	17 (13–20)	IV r-tPA	98	66.3 (±11.3)¶	17 (13–19)
ESCAPE^39^†	M.T. ± IV r-tPA	165	71 (60–81)‡	16 (13–20)	±IV r-tPA	150	70 (60–81)‡	17 (12–20)
THRACE^40^†	IV r-tPA ± M.T.	200	66 (54–74)‡	18 (15–21)	IV r-tPA	202	68 (54–75)‡	17 (13–20)
THERAPY^41^*	IV r-tPA ± M.T.	55	67 (±11)¶	17 (13–22)	IV r-tPA	53	70 (±10)¶	18 (14–22)
PISTE^42^	IV r-tPA ± M.T.	33	67 (±17)¶	18 (6–24)§	IV r-tPA	32	64 (±16)¶	14 (6–29)§
EASI^43^*	IV r-tPA ± M.T.	40	74 (62.7–80)‡	18 (13–21.75)	IV r-tPA	37	71 (59–79)‡	20 (12–23)

*Enrolment was halted early after positive results for thrombectomy were reported from other similar trials.

†Trial stopped early due to efficacy.

‡Median (IQR).

§Median (±range).

¶Mean (±SD).

IV r-tPA, intravenous recombinant tissue-type plasminogen activator; IA r-tPA, intra-arterial recombinant tissue-type plasminogen activator; MT, mechanical thrombectomy; NIHSS, National Institutes of Health Stroke Scale; IQR, interquartile range; SD, standard deviation.

**Table 3:** Effect of mechanical thrombectomy compared with best medical therapy on good functional outcome (modified Rankin Score≤2* at 90 days)

Trial	Mechanical thrombectomy	Best medical therapy	Adjusted OR (95% CI) p value
MR CLEAN^35^	76 (32.6)	51 (19.1)	2.16 (1.39–3.38)
REVASCAT^36^	45 (43.7)	29 (28.2)	2.1 (1.1–4.0)
EXTEND 1A^37^	25 (71)	14 (40)	4.2 (1.4–12) p=0.01
SWIFT-prime^38^	59 (60)	33 (35)	1.70 (1.23–2.33) p<0.001
ESCAPE^39^	87 (53.0)	43 (29.3)	1.7 (1.3–2.2)
THRACE^40^	106 (53)	85 (42)	1.55 (1.05–2.30) p=0.028†
THERAPY^41^	19 (38)	14 (30)	1.4 (0.60–3.3) p=0.55
PISTE^42^	17 (57)	10 (35)	4.92 (1.23–19.69) p=0.021‡
EASI^43^	20 (50)§	14 (38)¶	p=0.36

Figures are numbers of patients achieving a good functional outcome at 90 days after stroke (%).

*This corresponds to slight or no residual disability as a result of the stroke.

†Value at 30 days.

‡Per protocol population analysis.

§19/35 anterior circulation, 1/5 posterior circulation.

¶14/32 anterior circulation, 0/5 posterior circulation.

OR = odds ratio.

Powerful evidence for the safety and efficacy of mechanical thrombectomy comes from the ‘Highly Effective Reperfusion Evaluated in Multiple Endovascular Stroke Trials’ (HERMES) collaboration meta-analysis of the first five positive studies.^44^ By pooling individual data from 1287 patients, the meta-analysis could also investigate the efficacy of thrombectomy in subgroups that were too small to investigate in the individual trials. HERMES showed that the proportions of patients achieving a good (independent) functional outcome (mRS 0–2 at 90 days) were 46.0% (mechanical thrombectomy) vs 26.5% (best medical treatment). IV r-tPA was given to 83% of patients in the thrombectomy population and 87% of those in the control population. The number needed to treat for patients to achieve a reduction of 1 or more points on mRS was 2.6. Reassuringly, mortality at 90 days and risk of symptomatic intracerebral haemorrhage did not differ between patients receiving IV r-tPA and thrombectomy versus IV r-tPA alone. The benefit remained in subgroups of patients >80 years of age and those who did not receive IV r-tPA. Thrombectomy led to consistent benefit across National Institutes of Health Stroke Scale (NIHSS) scores, from milder to more severe strokes. Although there was no statistical heterogeneity of effect by the degree of early brain ischaemia measured by the Alberta Stroke Programme Early CT score (ASPECTS), there was clear benefit only for ASPECTS >5 (indicating a limited extent of early ischaemic tissue injury). However, there were only a few patients with ASPECTS <5 included. Other recent meta-analyses have confirmed the key findings from HERMES.^45–47^

Based on evidence from these trials, updated practice guidelines were rapidly published in the USA,^48^ Canada,^49^ Europe^50^ and in the UK,^51 52^ recommending that mechanical thrombectomy should be provided to patients with occlusion of the internal carotid artery or proximal middle cerebral artery who have received treatment with IV r-tPA within 4.5 hours of onset^53^ and who can undergo the procedure (arterial puncture) within 6 hours of symptom onset.

A further meta-analysis of the five studies^54^ showed improved outcomes when thrombectomy was performed up to 7.3 hours after symptom onset, in patients satisfying imaging criteria for the randomised trials, but there was still clearly greater benefit with faster intervention (<2 hours). Patients with moderate infarct core volumes (ASPECTS 7–8) had a shallower decline in benefit with longer symptom onset to reperfusion than patients with minor infarct core volumes (ASPECTS 9–10). The important message here is that, just as for IV r-tPA, speed of delivery of mechanical thrombectomy is key to achieving the best possible outcomes.  However, the time window for treatment may be longer for those with smaller irreversibly damaged ischaemic core.

## How to select patients

The decision to proceed with mechanical thrombectomy should be made by a physician trained in the diagnosis and treatment of stroke, in conjunction with a neurointerventionist who has the relevant brain and arterial imaging available for review. It is essential to have rapid, expert clinical assessment—for stroke diagnosis, localisation, severity stratification (NIHSS) and assessment of pre-stroke functional status (modified Rankin score) and comorbidities—and adequate brain and vascular imaging acquisition (typically CT and CT angiography) and interpretation. It is crucially important to have interaction, discussion and teamwork between stroke physician and neurointerventionist to make what are often complex and time-sensitive decisions. Extracranial vessel imaging (easily obtained with the same CT angiogram) is essential to determine the feasibility of access to the target artery occlusion. The selection criteria applied in practice should parallel those of the successful trials, including the following:

- documented large vessel anterior circulation occlusion (middle cerebral artery, M1 or internal carotid artery)
- significant clinical deficit at the time of treatment (this might be NIHSS>5 or a lower score that is functionally significant for the patient; note that even mild deficit from proven large vessel occlusion has a high risk of clinical deterioration)
- lack of extensive early ischaemic change (those with ASPECTS more than 5 on plain CT clearly benefit)
- pre-stroke functional status and lack of serious comorbidities indicating potential to benefit from treatment (note that age>80 years alone is NOT a contraindication to treatment)
- treatment with intravenous thrombolysis within 4.5 hours (although patients ineligible for intravenous thrombolysis due to bleeding risk were also included in some of the trials and might also reasonably be offered treatment)
- thrombectomy can be performed within 6 hours
- good collateral circulation (though benefit in patients with poor collaterals remains uncertain).

Areas of remaining uncertainty include patients with more distal occlusions (eg, M2); there was no statistical evidence of treatment effect heterogeneity in patients with M2 occlusion, but only 94 such patients were included in the clinical trials. Patients with substantial symptoms and technically accessible occlusions in proximal M2 might thus be reasonable to treat, but we need more evidence. There is also still only limited evidence for thrombectomy in basilar occlusion. The role of more advanced imaging also remains to be defined (beyond the mandatory CT and CT angiography, eg, CT perfusion, MRI DWI and PWI, which can more accurately define ischaemic core volume as well as potentially salvageable brain). Nevertheless, good outcomes have been achieved in the Netherlands and the UK using pragmatic CT angiogram-based patient selection (MR CLEAN and PISTE).

## How it is done

### Devices, technique and clot types

**Figure 1:**
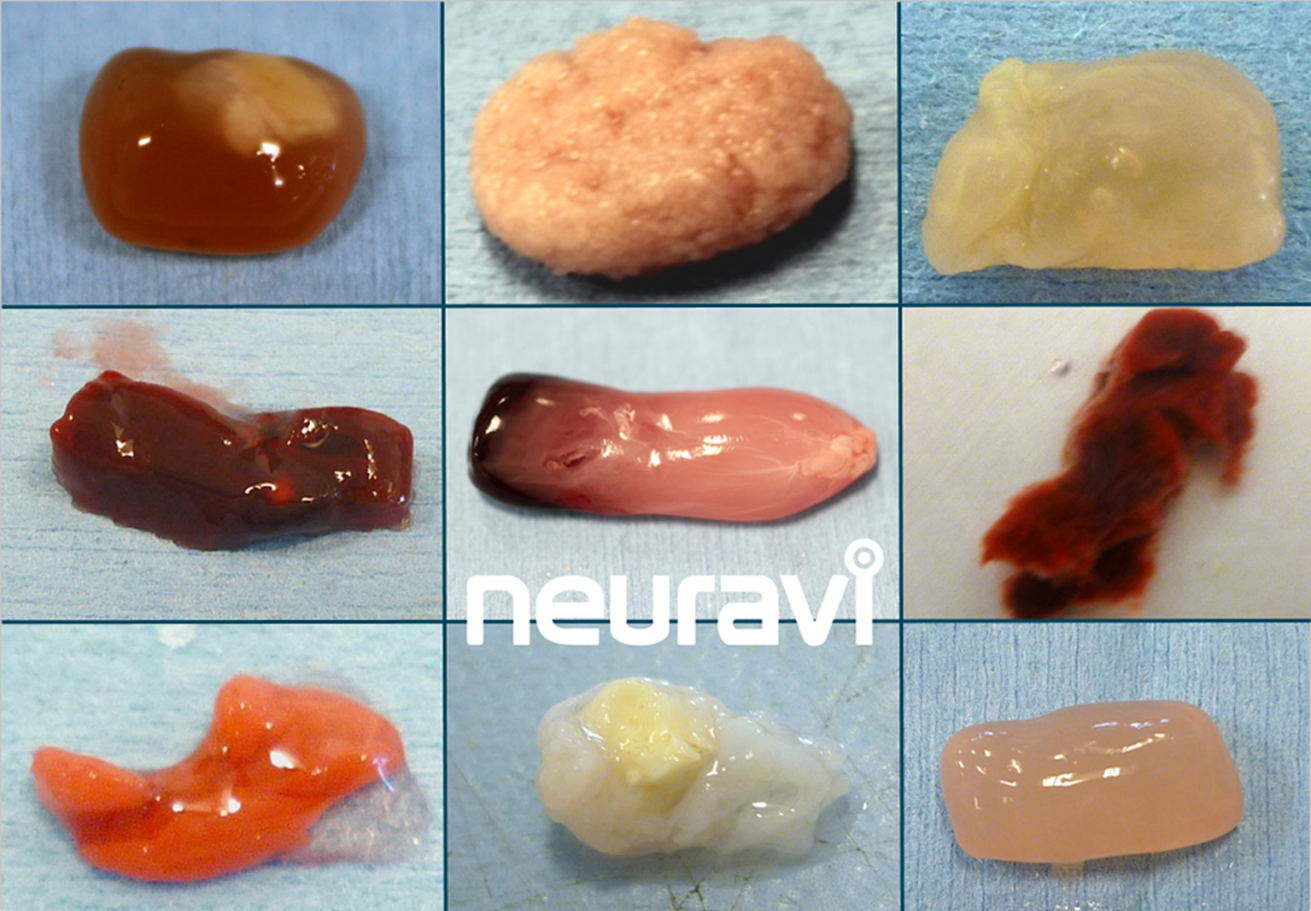
A range of different clot types, which have different physical properties, potentially requiring a range of thrombectomy techniques. These are experimental clot analogues, primarily from ovine blood. Image provided courtesy Neuravi.^84^

After the positive randomised trials, the Solitaire FR stent retriever device became the benchmark for mechanical thrombectomy. However, rapid and safe recanalisation and reperfusion of brain is the key factor, rather than any specific device or technique; there are multiple options available. In addition to the primary device, many supplementary devices and techniques are used, for example, balloon guide catheters, intermediate catheters and suction pumps versus manual aspiration, etc. Moreover, the variable underlying mechanisms and anatomy of large vessel occlusions may challenge the standard approach. There is a vast range of thrombus types (traditionally considered as ‘red’ or fibrin-rich (classically thought most likely to be cardioembolic) and ‘white’ or platelet-rich (classically thought most likely to result from atherosclerotic plaques). This dichotomy is now recognised to be an oversimplification; figure 1 shows a range of different potential clot types, which have different physical properties potentially requiring a range of thrombectomy techniques to optimise recanalisation; for example, friction properties (‘stickiness’) might relate to the ratio of fibrin to red blood cells.

The issue of general anaesthetic versus awake thrombectomy remains controversial, with little evidence to guide the decisions (see below). Our current approach is to proceed with the patient awake using local anaesthetic and analgesia and support from an anaesthetist. However, in severe dominant hemisphere internal carotid artery and middle cerebral artery occlusions, the patients may be very agitated and confused making the procedure difficult and unsafe; under these circumstances, it is entirely appropriate to recourse to general anaesthesia.

**Figure 2:**
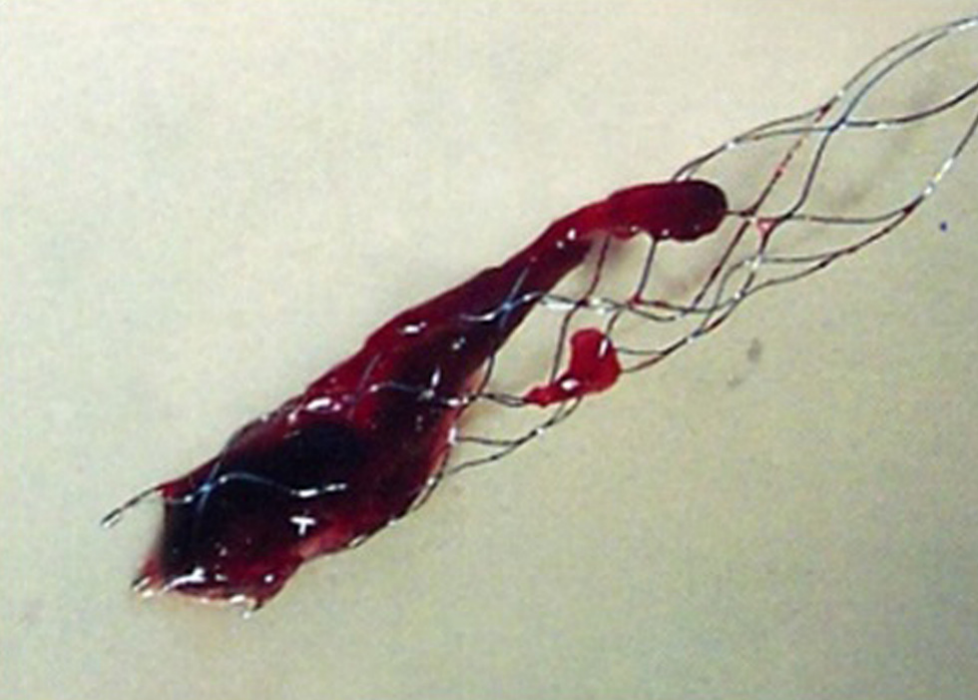
Freshly removed clot enclosed in a stent retriever device.

Normally, after femoral arterial puncture, a large (8Fr) guide catheter is navigated into the internal carotid artery, within which are an intermediate (5–6Fr) catheter (which is directed to the circle of Willis) and a microcatheter, which must be navigated through to the clot over a microguidewire. The microwire is removed, allowing the stent retriever to slide through the microcatheter to emerge inside the clot, where it opens like a stent (but remains attached to its pusher wire); once integrated with the clot, the device is pulled back into the intermediate catheter, to which suction is simultaneously applied. A balloon guide, forming a cuff around the guide catheter, may be used to stop forward flow and reduce the chance of embolising fragments of the clot distally or into other territories; when using such a guide, the intermediate catheter may be omitted. Figure 2 shows freshly removed clot in a stent retriever device.

An increasingly popular approach is to attempt to aspirate the clot directly into an intermediate catheter, which has become feasible with the advent of large lumen catheters that can safely be navigated into the M1 segment of the middle cerebral artery and beyond. The key here is to choose a catheter with a lumen approaching the size of the vessel where the clot is lodged, allowing the clot to be suctioned in its entirety; if this fails, a stent retriever may easily be deployed through the initial system.

Common challenges are tandem occlusions of cervical internal carotid artery and intracranial vessels and fixed intracranial stenosis, which can limit endovascular access; patients often have tortuous and ectatic large vessels, with an ‘unfolded’ aortic arch or redundant cervical carotid loops. The primary access point is the common femoral artery, but the radial or brachial approach is an alternative for those with aorto-ilial-femoral disease; the direct carotid approach has also been proposed but remains unpopular due to safety concerns. When there is a tandem occlusion secondary to carotid disease in the neck, the interventionist has to decide which lesion to treat first and whether to deploy a carotid stent or just angioplasty any stenotic lesions. There is currently no evidence or guideline to direct this decision. Similarly with fixed intracranial stenosis, when the clot has been removed revealing a tight (and possibly unstable) stenotic plaque, the choice is between angioplasty and stenting. In both of these circumstances, there is a need to maintain dual antiplatelet blockade if a stent is left *in situ*, which might exacerbate any haemorrhagic complications after stroke. A systematic review of 32 studies included 1107 patients with internal carotid artery occlusions found that acute stenting of occlusions of the extracranial internal carotid artery resulted in a higher recanalisation rate (87% vs 48%, p=0.001) and better outcomes (68% vs 15%, p<0.001) as well as lower mortality (18% vs 41%, p=0.048) when compared with intra-arterial thrombolysis.^55^

Recent cohort studies suggest that tandem stenosis/occlusions of the internal carotid/middle cerebral arteries can be treated with acute stenting of the extracranial internal carotid and stent retriever mechanical thrombectomy in the middle cerebral artery with acceptable risk profile, but further research of the safety profile and benefit of this approach is needed.^56–60^

### Complications and how they are managed

Complications of endovascular procedures can result from direct device-related vascular injury, vascular access and the use of radiological contrast media. Stent retriever devices are generally safe^61^ with lower complication rates than first-generation devices. The most common complications include the following: vessel perforation,^62–64^ which occurred in 1.6% patients in the five recent positive endovascular trials (range 0.9%–4.9%); symptomatic intracranial haemorrhage (3.6%–9.3%); subarachnoid haemorrhage (0.6%–4.9%); arterial dissection (0.6%–3.9%); emboli to new territories (1.0%–8.6% in randomised controlled trials); vasospasm; and vascular access site complications (including dissection, pseudoaneurysm, retroperitoneal haematoma and infection). The overall procedural complication rate from recent randomised controlled trials is in the range of 15%, but it must be emphasised that many do not adversely affect clinical outcome. Stent retriever detachment^65 66^ is an uncommon complication (about 2%–3% with first-generation Solitaire FR device, but anecdotally much lower with the latest versions).

**Figure 3:**
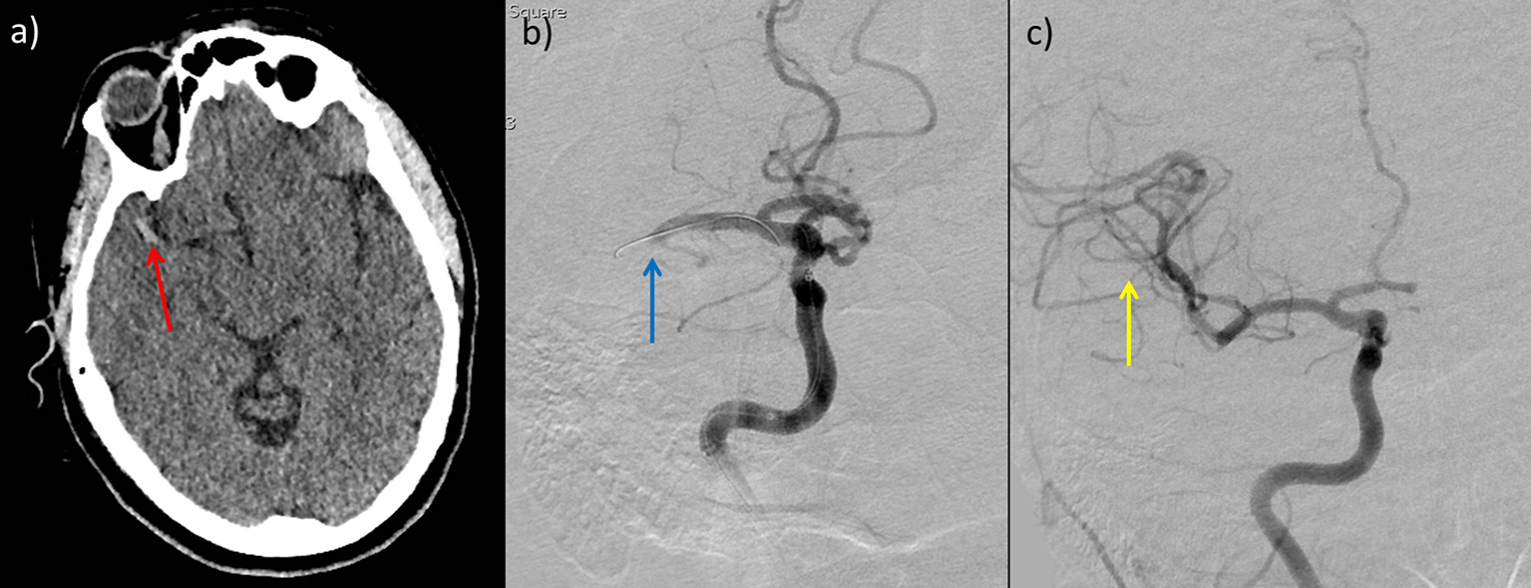
Plain CT scan of head (a) and prethrombectomy (b) and post-thrombectomy (c) digital subtraction angiograms in a 49-year-old woman with sudden onset left hemiparesis and confusion. Plain CT scan of head shows hyperdense clot in the right middle cerebral artery (red arrow) and early perisylvian loss of grey–white matter differentiation. Prethrombectomy digital subtraction angiogram shows occluded right proximal middle cerebral artery (blue arrow). The catheter is visible passing through the occlusion. Post-procedure imaging shows good filling of all middle cerebral artery branches (yellow arrow). There was complete resolution of neurological signs and symptoms following aspiration thrombectomy.

**Figure 4:**
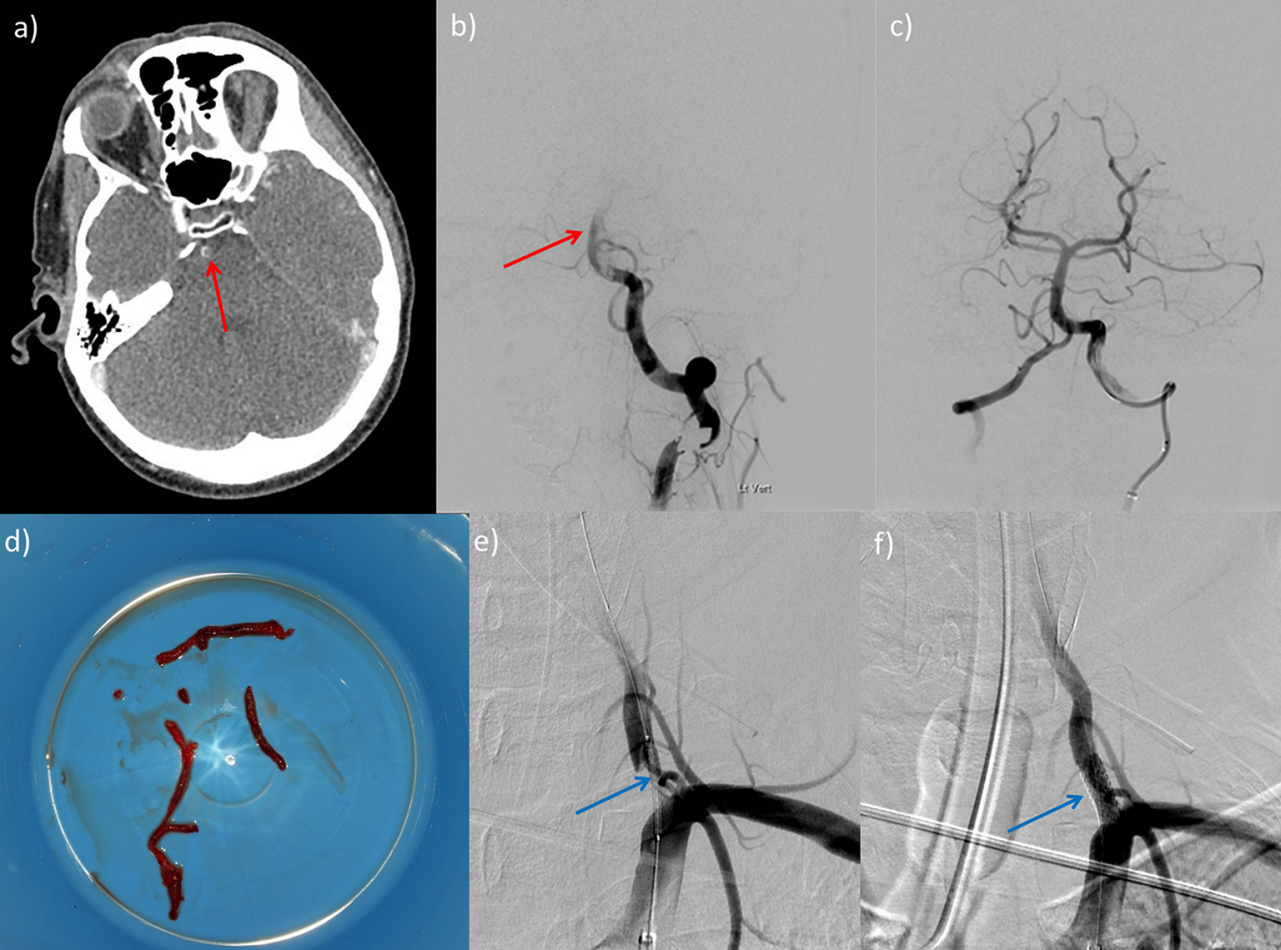
Plain CT scan of head (a) and prethrombectomy (b) and post-thrombectomy (c, e, f) digital subtraction angiograms in a 58-year-old man with a short history of visual symptoms and vertigo followed by a rapid drop in conscious level. Plain CT scan of head (a) shows thrombus in the basilar artery (red arrow) with complex plaque at the vertebral artery origin, confirmed on digital subtraction angiography (b). Following successful thrombectomy (c), with removal of a large cast of thrombus (d) by aspiration, a stent was deployed across the unstable stenotic plaque at the vertebral artery origin (blue arrows, e and f). Basilar thrombi can often be removed in bulk like this, possibly because of their physical composition.

**Figure 5:**
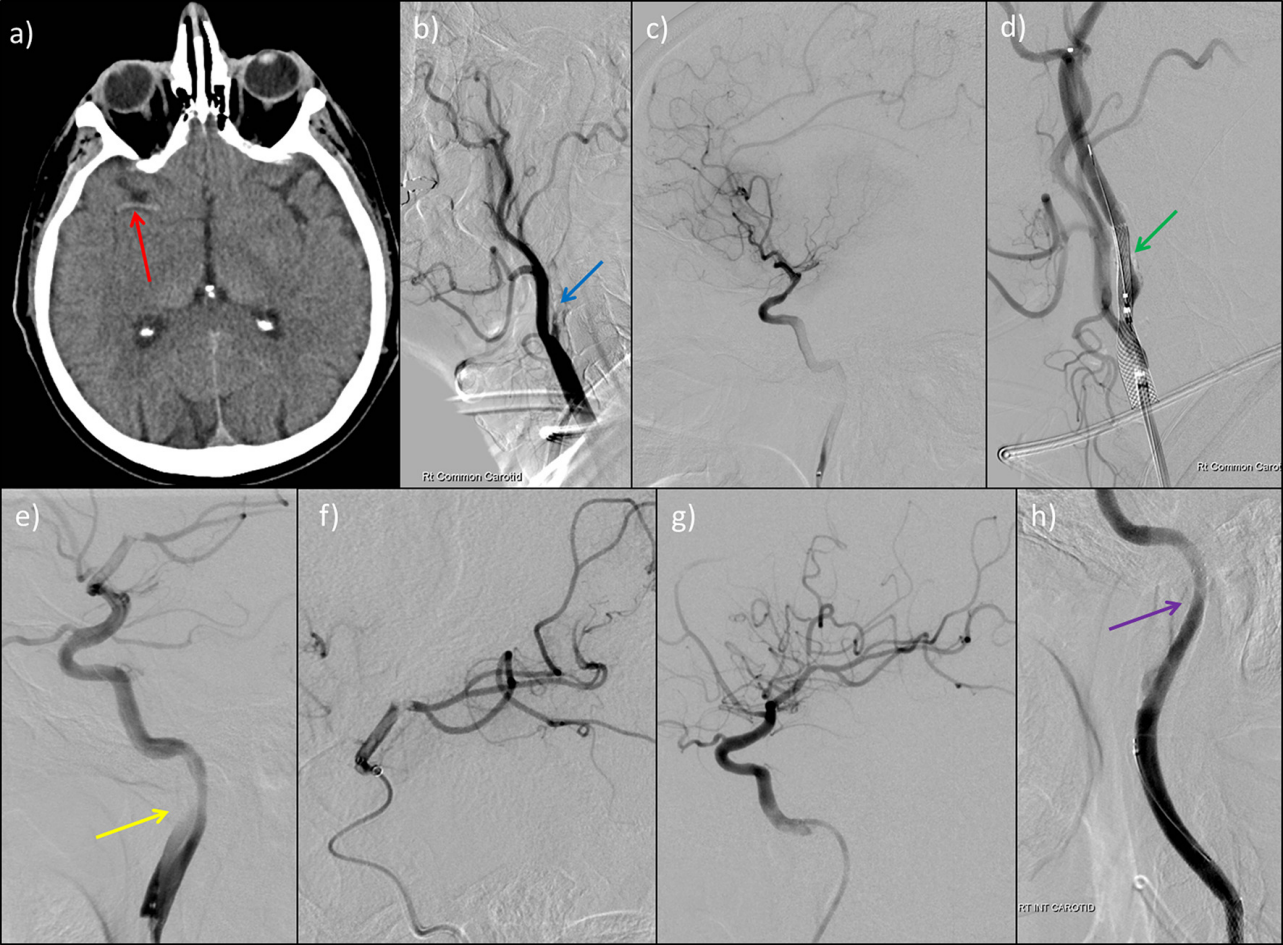
Plain CT scan of head (a) and prethrombectomy (b, c), during thrombectomy (d, e, f) and post-thrombectomy (g, h) digital subtraction angiogram images in a 61-year-old man who presented with a 10 min seizure, followed by left-sided weakness and neglect. Plain CT scan of head shows hyperdense thrombus in the right middle cerebral artery (red arrow, a) with angiogram identifying a critical stenosis of the internal carotid artery origin (blue arrow, b). We performed middle cerebral artery thrombectomy using stent retriever technique (e and f). An internal carotid artery stent was inserted (green arrow, d) complicated by an iatrogenic dissection (yellow arrow, e) necessitating stenting (purple arrow, h).

The key strategy to minimise complications is obvious and simple: for thrombectomy to be only performed in high-volume centres by trained physicians competent in intracranial endovascular procedures and undertaking them regularly to maintain skills, as recommended by various multidisciplinary guidelines.^51 52^ Mechanical thrombectomy should only be performed by a multidisciplinary team operating within comprehensive stroke centres with adequate neurointerventional procedural volumes (eg, >200 per annum), of which a reasonable proportion are mechanical thrombectomy and undertaking regular assessment/audit of technical and clinical results, process times and complications. When complications do occur, the immediate availability of neurocritical care and (less frequently required) neurosurgical support are mandatory and may be lifesaving. Figures 3–5 present three examples of thrombectomy procedures to demonstrate some of the potential complexities of the procedure.

### How are patients cared for before, during and after the procedure

Based on the published trial evidence, treatment should ideally be undertaken in major neurointerventional centres with well-functioning hyperacute stroke units and with rapid access to neurosurgical and neurointensive care facilities. Currently intravenous thrombolysis is typically administered as soon as the diagnosis of ischaemic stroke is made, if the patient is within 4.5 hours and there are no contraindications. Evidence for ‘primary’ thrombectomy without intravenous thrombolysis remains limited but there are trials both ongoing and proposed.

### Anaesthesia

The use of general versus local anaesthesia (conscious sedation) currently varies; each strategy has potential advantages. General anaesthesia reduces subject distress and movement, and it can make the technical aspects easier; on the other hand, conscious sedation allows continuous neurological monitoring for complications, and it avoids any potential hazard of general anaesthetic agents. Retrospective data comparing general with local anaesthesia during the procedure found that general anaesthesia, often associated with systolic blood pressure<140 mm Hg, was associated with a poor functional outcome (mRS >2) at 90 days.^67^ However, a recent single-centre randomised trial in 150 patients with acute ischaemic stroke found similar early (24 hours) outcomes (measured as NIHSS change) from general anaesthesia with intubation or conscious sedation without intubation during thrombectomy.^68^ Moreover, two studies presented at the 3rd European Stroke Organisation Conference (ESOC) in 2017 (GOLIATH and ANSTROKE) both suggested that general anesthaesia and  conscious sedation are equally safe. Thus, either approach currently seems reasonable, and the choice should be guided by careful consideration of each individual patient (eg, agitation, neurological or haemodynamic stability, ease of vascular access to the target lesion, etc). Ongoing trials of general anaesthesia versus conscious sedation should provide a clearer answer.

### Blood pressure

Based on several neutral randomised trials of blood pressure lowering, guidelines suggest that lowering blood pressure in acute ischaemic stroke should be postponed, at least for a day or two, unless it is severely elevated (>220/120 mm Hg, or >200/100 mm Hg with acute kidney injury, aortic dissection, cardiac ischaemia, hypertensive encephalopathy or pulmonary oedema).^69^ Following thrombectomy, medical and nursing teams are often uncertain how to manage blood pressure. However, there is limited evidence to guide how blood pressure should be managed before, during and after thrombectomy. It has been suggested that the poorer functional outcome (mRS>2) at 90 days associated with general anaesthesia might relate to the generally lower blood pressure (usually <140 mm Hg systolic), but this study could not account for confounding factors.^68^ Given the lack of evidence, we currently recommend maintaining blood pressure within a physiological range (typically 110–160 mm Hg systolic) in a high dependency (eg, neurocritical care) setting following thrombectomy. Specific situations (eg, critical extracranial or intracranial stenosis with haemodynamic failure or post-procedure intracranial bleeding) may require different blood pressure targets.

### Antithrombotic treatments

There is little evidence on optimum antithrombotic treatment during and after thrombectomy. Urgent anticoagulation is not generally recommended in acute ischaemic stroke due to the risk of intracranial haemorrhage. Aspirin is not recommended within 24 hours of IV r-tPA but should be started orally (or via nasogastric tube) within 24–48 hours after stroke onset. Randomised trials and registries do not give consistent data or recommendations regarding antithrombotic use in mechanical thrombectomy. Some units give a single procedural dose of heparin, but they avoid antiplatelet medication or further anticoagulation for 24 hours from stroke symptom onset, and they suggest  follow-up brain imaging with CT or MRI to exclude haemorrhagic complications, but practice varies. If we do not deploy a stent, after 24 hours and satisfactory clinical progress and follow-up imaging to exclude significant haemorrhage, we then give aspirin 300 mg for up to 2 weeks, followed by long-term secondary prevention. This will depend on stroke mechanism: usually clopidogrel or aspirin for non-cardioembolic and oral anticoagulation for atrial fibrillation or other cardioembolic sources. If we do deploy a stent, we recommend acutely starting treatment with aspirin and clopidogrel (or equivalent) dual antiplatelet therapy for at least 3–6 months. For stents in patients requiring anticoagulation we generally switch to a single antiplatelet agent for long-term secondary prevention.

In the hyperacute clinical setting of mechanical thrombectomy, it is easy to forget the important task, as in all patients with acute stroke, of working out the likely causative processes and mechanism(s) to optimise preventive treatment. For example, this might involve specific investigations for arterial dissection, detailed cardiac structure and rhythm evaluation and investigations for thrombophilias or systemic disease.

## Challenges and the future

Thrombectomy with reperfusion ≤6 hours after symptom onset, alongside IV r-tPA, is clearly the new standard of care for the treatment of acute ischaemic stroke due to large vessel occlusion in the anterior circulation. The UK National Institute for Health and Care Excellence (NICE) now approves the use of mechanical thrombectomy in stroke.^70^ However, many challenges remain, including, crucially, its practical implementation.

### Can mechanical thrombectomy be delivered in the UK?

The PISTE trial (run in 10 English neuroscience centres) data confirm the generalisability of the compelling randomised trial results and show that thrombectomy can be safely and effectively delivered within the National Health Service (NHS).^42^ At the time of writing, only two UK centres offer 24 hour endovascular clot retrieval, with most others providing the service only during working hours. Modelling work based on Sentinel Stroke National Audit Programme (SSNAP) data, randomised controlled trials and other high-quality evidence indicates that, based on the current criteria and guidelines listed above, around 10% of all stroke admissions in the UK (around 9500 patients) would be eligible for thrombectomy annually. The great bulk of those would come from patients presenting to hospital within 4.5 hours of stroke onset. However, ongoing randomised trials may well expand those eligibility criteria further over the next 3–5 years, for example, for strokes in the 6–12 hour window, in stroke of unknown time onset and in mild strokes (NIHSS<6).

### What are the challenges in delivering thrombectomy as standard clinical practice in the UK?

Rapid access to appropriate imaging (non-contrast CT scan of head and CT angiogram at a minimum) is mandatory in selecting patients appropriate for endovascular treatment. Although this has been recently recommended in UK national guidelines,^71^ it is not yet standard care in all acute stroke centres, and so routine availability of CT angiography for acute stroke needs to be rapidly increased. Making CT angiography a routine acute stroke investigation can bring major gains in speeding up diagnostic pathways, as stroke teams and radiographers develop expertise and familiarity with the processes. Although there have been attempts to perform CT angiography only in more severe strokes (who are more likely to have a large vessel occlusion), patients with milder strokes commonly have large vessel occlusion (about 10% of those with NIHSS <6) with a high risk of clinical worsening.^72^ Once a potentially treatable large vessel occlusion is identified (which requires 24/7 access to trained neuroradiologists or stroke physicians), treatment must then be delivered quickly. In the positive trials detailed above, median time between symptom onset to femoral artery puncture was less than 4 hours; median time from symptom onset to recanalisation was 4 1/3 hours. This timeframe is currently a challenge in certain parts of the UK, particularly outside standard working hours, and will require innovation in local imaging acquisition and interpretation, as well as emergency transport services. Once a patient eligible for endovascular clot retrieval is identified, there should be no delay in transferring them to an appropriate centre; however, administration of IV r-tPA should not be delayed, since this is still the cornerstone of initial treatment (and given in around 90% of patients in the nine recently published clinical trials of thrombectomy).^73^ We need meticulous organisation and robust, well-audited care pathways to enable safe and rapid transfer. The two potential models for providing thrombectomy can be described as ‘*drip and ship*’ (initial transfer to a local stroke centre for diagnosis and intravenous thrombolysis, followed by rapid transfer to a specialist thrombectomy centre) and ‘*mothership*’ (transfer immediately to a specialist comprehensive stroke centre able to undertake thrombectomy and other required neuroscience support services). The optimal model will vary according to local geography including population density, transport infrastructure and distance from specialist centres able to deliver the treatment safely and effectively. ‘Drip and ship’ might be the more appropriate solution for more remote areas, while a ‘mothership’ model might be a good solution for urban city populations.

### How (and by whom) should thrombectomy be delivered?

Endovascular clot retrieval is safe, but only in experienced, appropriately trained, competent hands. It is a complex procedure requiring an experienced team to deliver, and it needs to be performed with great rapidity. Therefore, thrombectomy delivery will need to be centralised so that centres and teams can develop expertise quickly and maintain 24/7 services robustly and so that neurointerventionists can undertake a sufficient number of cases to maintain expertise. In the UK, published guidance on training and competencies for thrombectomy is helpful here, and it indicates that a caseload of at least 40 intracranial endovascular interventions per year is required to maintain competency in neurointervention.^74^ We need new ways of thinking about care delivery both before and after completion of training to expand the pool of skilled neurointerventionists. Although there has been considerable interest in whether thrombectomy could be safely delivered by other (non-interventional neuroradiology) specialties, the skills required to open cerebral arteries quickly, safely and effectively might not be generic across these other specialties; for example, although coronary and cerebral arteries are of similar calibre, brain arteries are more delicate with a thinner tunica media and adventitia, often with proximal ectasia and tortuosity, making navigation with a catheter both hazardous and challenging. Moreover, dealing with sudden neurological complications requires great skill and expertise in navigating the complex cerebral vasculature. Indeed, the best results in clinical trials and clinical practice were achieved by experienced neurointerventionists in high-volume centres.^75^ We need international mechanical thrombectomy registries to identify whether the real-world experience is commensurate with that seen in the positive clinical trials; SITS thrombectomy is one such registry.

### Is mechanical thrombectomy cost effective?

One study^76^ modelled the hyperacute management of stroke using intravenous thrombolysis and mechanical thrombectomy in the UK (compared with intravenous thrombolysis alone) using Markov simulations of estimated lifetime costs and quality-adjusted life years (QALYs), based on pooled outcome data of five randomised controlled trials. This study found an incremental cost per QALY gained of mechanical thrombectomy over a 20 year period of $11 651 (£7061). A more recent study that modelled the intervention in a US setting found an incremental cost-effectiveness ratio for endovascular treatment (compared with standard care) of $3110/QALY (about £2500 per QALY) in all simulations, although cost effectiveness was lower in more distal (M2) occlusion and with established ischaemic injury (ASPECTS score ≤5). Both of these studies show that the cost of mechanical thrombectomy is well below the frequently applied £30 000 per QALY threshold used by National Institute for Health and Care Excellence (NICE) to evaluate new treatments.

### Future research questions

There are many remaining questions regarding thrombectomy. We have few data on thrombectomy for basilar artery thrombosis; some registry data suggest that a high proportion of patients (68%) have a poor outcome (mRS>3), with no difference according to the use of intravenous thrombolysis or mechanical thrombectomy.^77^ As in the anterior circulation, recanalisation is a key prognostic factor: a recent meta-analysis of 45 observational studies (n=2056) of reperfusion versus no reperfusion of acute basilar occlusion showed a number needed to treat to decrease death or dependency of 3.^78^ Small single-centre studies reported good functional outcomes following basilar thrombectomy, ranging from 30% to 48%.^79–82^ The time window may be longer for basilar thrombosis (possibly up to 12–24 hours), perhaps relating to the tissue properties, clot composition and haemodynamics of the collateral vascular supply in the posterior circulation. We need randomised trials of thrombectomy, but it might be challenging because of lack of clinical equipoise (given the clear benefits of thrombectomy in the anterior circulation and the devastating outcome from untreated basilar thrombosis); one trial of treatment within 6 hours is underway.^83^

The optimum form of imaging acquisition and processing (to determine the extent of ischaemic core, potentially salvageable tissue, collateral supply, etc.) requires further study. Is MRI better than CT? Is perfusion imaging required, or will collateral assessment and APSECTS suffice? This is a very important question for thrombectomy implementation as imaging triage is likely to be critical in the ‘drip and ship’ service model in particular. For many logistic reasons, it would be preferable to secondarily transfer for thrombectomy only those patients who are very likely to benefit. We also need to know whether all patients need advanced brain imaging or just some, and if some, who? The PISTEai (advanced imaging) trial is proposed in the UK to answer some of these questions.

Another critical question is whether we should use advanced imaging in more delayed (including ‘wake-up’ stroke) presentations. Trials are ongoing, including POSITIVE (6–12 hour time window with appropriate image selection). The DAWN trial (6–24 hour time window, including wake-up stroke) selected patients with substantial clinical deficit but a small ischaemic core on CT-perfusion imaging and randomised them to mechanical thrombectomy with the Trevo device, or to medical therapy alone. The DAWN trial was stopped early on 9/3/2017 after ~200/500 patients had been recruited. Data from the DAWN trial presented at the European Stroke Organisation Conference (ESOC) in May 2017 indicated that at 90 days, 48.6% of patients in the intervention arm achieved functional independence, compared to 13.1% in the control medical therapy arm. Evidence of an extended time window for mechanical thrombectomy potentially means that more stroke patients might be eligible for endovascular treatment. However, these data should not detract from the key message that the most rapid treatment possible remains the key aim to optimise outcomes for all reperfusion therapies in acute stroke.

There are also trials proposed of thrombectomy in patients with milder stroke. The role of direct thrombectomy (without intravenous thrombolysis) also remains to be defined in randomised controlled trials.

Many of these pressing remaining questions about mechanical thrombectomy will probably be answered within the next 3–5 years. All patients undergoing mechanical thrombectomy should also be prospectively included in registries to obtain further evidence on effectiveness and safety in ‘real world’ practice.

## Conclusion

Mechanical thrombectomy is a highly successful, safe and cost-effective treatment for patients with large artery occlusive stroke. It is therefore a ‘no brainer’ that the UK NHS and other healthcare systems need to deliver it as soon as practicable. However, that will inevitably require reorganisation of UK stroke services and that will require substantial investment, great attention to care pathways and extensive cooperation between services including ambulance services and hospitals.

**Key points**

- Thrombectomy for anterior circulation stroke due to proven proximal major vessel (carotid or M1) occlusion within 6 hours of stroke onset is safe and highly effective, and sets the new standard of care 
- In a meta-analysis of randomised trials, the proportions of patients achieving a good (independent) functional outcome (mRS 0–2 at 90 days) were 46.0% (mechanical thrombectomy) vs 26.5% (best medical treatment); most patients also received intravenous thrombolysis 
- Favourable outcome from mechanical thrombectomy is strongly time dependent ('time is brain'), with the best results achieved when there is no evidence of extensive early ischaemic brain injury (e.g. ASPECTS score >5); if good recanalisation is achieved within 4.5 hours, the absolute rate of good functional outcome is 61%
- Complications of endovascular procedures can follow device-related vessel injury (perforation, dissection, subarachnoid haemorrhage), vascular access or radiological contrast media
- Thrombectomy must be delivered by appropriately trained interventionists
- The next challenge is in delivering the treatment across healthcare systems; the optimal solution (eg, 'drip and ship' versus 'mothership') may differ according to geography and population density
